# Synergistic effect of YOD1 and USP21 on the Hippo signaling pathway

**DOI:** 10.1186/s12935-023-03078-3

**Published:** 2023-09-24

**Authors:** Sang-Soo Park, Kwang-Hyun Baek

**Affiliations:** 1 Department of Biomedical Science, CHA University, 335 Pangyo-Ro, Bundang-Gu, Seongnam-Si, Gyeonggi-Do 13488 Republic of Korea; 2 Department of Bioconvergence, CHA University, 335 Pangyo-Ro, Bundang-Gu Seoungnam-Si, Gyeonggi-Do, 13488 Republic of Korea

**Keywords:** Bioinformatics, Cell proliferation, Cell signaling, Deubiquitination, Post-translational modification, Ubiquitin‐dependent protease

## Abstract

**Background:**

Deubiquitinating enzymes (DUBs) comprise a family of proteases responsible for cleaving the peptide or isopeptide bond between ubiquitin and its substrate proteins. Ubiquitin is essential for regulating diverse cellular functions by attaching to target proteins. The Hippo signaling pathway plays a crucial role in controlling tissue size, cell proliferation, and apoptosis. In a previous study, we discovered that YOD1 regulates the Hippo signaling pathway by deubiquitinating the neural precursor cell expressed developmentally down-regulated protein 4 (NEDD4), an E3 ligase of large tumor suppressor kinase 1 (LATS1). Here, our aim was to investigate potential substrates of YOD1 implicated in the Hippo signaling pathway.

**Methods:**

We employed various bioinformatics tools (BioGRID, STRING, and Cytoscape) to identify novel potential substrates of YOD1. Furthermore, we used western blotting, co-immunoprecipitation (co-IP), glutathione S-transferase (GST) pull-down, immunocytochemistry (ICC) assays to investigate cellular interactions. To evaluate cell proliferation, we performed cell counting kit-8 (CCK-8), wound healing, colony forming, and flow cytometry assays using A549, HEK293T, and HeLa cells. Additionally, we assessed the expression levels of YAP and p-YAP in A549, HEK293T, and HeLa cells through western blotting.

**Results:**

Our investigations revealed that YOD1 interacts with ubiquitin-specific proteases 21 (USP21), a DUB involved in the Hippo signaling pathway, and deubiquitinates the microtubule-affinity regulating kinase (MARK). Intriguingly, YOD1 and USP21 mutually deubiquitinate each other; while YOD1 regulates the protein stability of USP21, USP21 does not exert a regulatory effect on YOD1. Moreover, we observed the synergistic effect of YOD1 and USP21 on cell proliferation through the modulation of the Hippo signaling pathway.

**Conclusions:**

Our study revealed multiple cellular interactions between YOD1 and USP21. Moreover, our findings suggest that the combined activities of YOD1 and USP21 synergistically influence cell proliferation in A549 cells by regulating the Hippo signaling pathway.

**Supplementary Information:**

The online version contains supplementary material available at 10.1186/s12935-023-03078-3.

## Background

The Hippo signaling pathway plays a vital role in regulating cell proliferation, survival, and apoptosis [1]. Recent studies have uncovered the involvement of Hippo signaling pathway in oncogenesis, and its dysregulation contributes to tumor initiation and progression [2–4]. The Hippo signaling pathway can be activated by various stimuli, such as cell density, mechanical and stress signals, or G protein-coupled receptors (GPCRs) [1]. Upon activation, the Hippo kinase is phosphorylated, followed by phosphorylation of Sav, large tumor suppressor kinase 1/2 (LATS1/2), and Mob by mammalian Ste20-like kinases 1/2 (MST1/2). Subsequently, LATS1/2 phosphorylates yes-associated protein 1 (YAP)/transcriptional coactivator with PDZ-binding motif (TAZ), leading to their phosphorylation [1]. Phosphorylated YAP/TAZ interacts with 14-3-3, reducing their activity and preventing them from entering the nucleus. This results in the retention of YAP/TAZ in the cytoplasm and their subsequent degradation through the ubiquitin-mediated pathway [5]. Conversely, when the kinases are inactive, the dephosphorylated YAP/TAZ translocates into the nucleus and induces gene transcription related to cell proliferation [5].

Ubiquitination is a vital post-translational modification (PTM) that regulates various cellular processes. The enzymatic cascade for ubiquitination involves three steps: E1 (ubiquitin-activating enzyme), E2 (ubiquitin-conjugating enzymes), and E3 (ubiquitin-protein ligases) [6]. Ubiquitin is a 76 amino acid protein that has seven lysine residues (K6, K11, K27, K29, K33, K48, and K63) and one methionine site (M1) [7]. Polyubiquitin chain linkages can be formed on any of these residues by conjugating another ubiquitin [7]. The choice of the lysine residue determines the fate of the substrate [8]. K6-linked polyubiquitin responds to mitophagy and DNA damage [9, 10]; K11-linked polyubiquitin controls the cell cycle, proteasomal degradation, protein stability, mitophagy, trafficking, and endoplasmic reticulum-associated protein degradation [11]; K27-linked polyubiquitin activates kinases and regulates DNA repair [12]; K29-linked polyubiquitin modifies kinases, promotes proteasomal/lysosomal degradation, regulates proteotoxic stress response, and affects the cell cycle [13, 14]; K33-linked polyubiquitin induces DNA damage, modifies kinases, activates innate immunity, induces autophagy, and regulates protein trafficking [9, 15, 16]; K48-linked polyubiquitin regulates proteasomal degradation [17], whereas K63-linked polyubiquitin induces signal transduction, activates kinases, and regulates autophagic degradation [18, 19].

Deubiquitinating enzymes (DUBs) are enzymes that play a critical role in the disassembly of ubiquitin on target substrates and polyubiquitin chains [20]. YOD1, also known as OTU1 and OTUB2, is a DUB that regulates various intracellular processes, such as cell cycle progression, transcriptional activation, and signal transduction [21]. In a recent study, we discovered that YOD1 interacts with neuronal precursor cell-expressed developmentally downregulated 4 (NEDD4), a ubiquitin E3 ligase that induces proteasomal degradation of LATS1 [22, 23]. Moreover, YOD1 is responsible for deubiquitinating NEDD4 and inhibiting NEDD4-mediated proliferation via the Hippo signaling pathway [22]. Another crucial role of YOD1 in the Hippo signaling pathway is to deubiquitinate ITCH, a member of the NEDD4 family of ubiquitin E3 ligases, as well as a ubiquitin E3 ligase of LATS [21].

To identify potential YOD1 substrates, we employed bioinformatics tools, including BioGRID (https://thebiogrid.org/), STRING (https://string-db.org/), and Cytoscape (https://cytoscape.org/), to analyze protein–protein interactions (PPIs). Among the putative substrates of YOD1, ubiquitin-specific peptidase 21 (USP21) is involved in both the Hippo signaling pathway and the ubiquitin–proteasome system (UPS). USP21 inhibits tumor growth by deubiquitinating and stabilizing microtubule affinity-regulating kinase (MARK), which restricts YAP/TAZ activity [24].

Over the years, several studies have demonstrated the role of DUBs in regulating components of the Hippo signaling pathway. However, there has been no study reporting an interaction between two DUBs involved in the Hippo signaling pathway, resulting in a synergistic effect. In this study, we provide evidence that two enzymes involved in the Hippo signaling pathway, namely YOD1 and USP21, interact with each other. Furthermore, YOD1 and USP21 deubiquitinate each other, with YOD1 upregulating USP21 protein stability, but not the reverse. We further investigate the synergistic effect of YOD1 and USP21 on cell proliferation and the Hippo signaling pathway.

## Materials and methods

### Plasmid construction and antibodies

*YOD1* was subcloned into the pcDNA3-6Myc vector using the forward primer 5ʹ-GAA TTC GGA TGT TTG GCC-3ʹ and reverse primer 5ʹ-CTC GAG TCA CAC TTC TCC-3ʹ. The pCMV3-Flag*-USP21* cDNA (HG13143-CF, SinoBiological, Beijing, China) was purchased, and *USP21* was subcloned into the pCS4-3Flag vector using the forward primer 5ʹ-ATG CCC CAG GCC TCT GAG-3ʹ and the reverse primer 5ʹ-TCA CAG GCA CCG GGG TGG-3ʹ. Deletion constructs of YOD1 and USP21 were subcloned into the pcDNA3-6Myc and pCS4-3Flag vectors, respectively, from the corresponding full-length cDNA. Site-directed mutagenesis was used to generate a catalytically inactive form of *YOD1* (C160S) and *USP21* (C221S). After PCR amplification, only the mutant form of YOD1 or USP21 was selected using the Dpn I enzyme (R054S, Enzynomics, Daejeon, Korea). Deletion constructs of YOD1 and mutants of *YOD1* (C160S) and *USP21* (C221S) were confirmed by direct sequencing (Cosmogenetech, Seoul, Korea).

The anti-HA (12CA5) and anti-Myc (9E10, CRL-1729, ATCC, Manassas, VA, USA) antibodies were acquired from hybridoma cell media. Anti-Flag (M185-3L, Sigma-Aldrich, St. Louis, MO, USA), anti-β-actin (sc-4778, Santa Cruz Biotechnology, Santa Cruz, CA, USA), anti-USP21 (sc-515911, Santa Cruz Biotechnology, Santa Cruz, CA, USA), anti-YOD1 (25370-1-AP, Proteintech, Santa Cruz, Rosemont, IL, USA), YAP (12395, Cell Signaling, Danvers, MA, USA), and p-YAP (4911, Cell Signaling, Danvers, MA, USA) antibodies were used for western blotting, GST pull-down, and immunoprecipitation (IP) assays.

### Cell culture condition, constructs, and transfection

HeLa cells, a human cervix adenocarcinoma cell line (CCL-2, ATCC, Manassas, VA, USA), and HEK293T cells, a transformed human embryonic kidney cell line CRL-11268, ATCC, Manassas, VA, USA), were cultured in Dulbecco’s Modified Eagle’s Medium (DMEM, 12800-017, Gibco, Grand Island, NY, USA) supplemented with 10% fetal bovine serum (FBS, 12483‑020, Gibco, Grand Island, NY, USA) and 1% antibiotic–antimycotic reagent (15240‑062, Gibco, Grand Island, NY, USA). A549 cells, a (human lung cancer cell line (CCL-185, ATCC, Rockville, MD, USA), were grown in RPMI-1640 medium containing 10% FBS (12,483‑020, Gibco, Grand Island, NY, USA) and 1% antibiotic–antimycotic reagent (15240‑062, Gibco, Grand Island, NY, USA). The cells were incubated in a 5% CO_2_ incubator at 37 °C. Transfection was performed using 10 mM polyethyleneimine reagent (PEI, 23966, Polysciences, Inc., Warrington, PA, USA) and 150 mM NaCl.

### Western blotting and IP

Western blotting was performed as previously described [25]. Briefly, harvested cells were lysed using a lysis buffer (50 mM Tris–HCl, 300 mM NaCl, 1 mM EDTA, 10% Glycerol, 1% Triton X-100), supplemented with 1% protease inhibitor cocktail (11697498001, Roche, Mannheim, Germany) and 1% phenylmethanesulfonyl fluoride (P7626, Sigma-Aldrich, St. Louis, MO, USA). After centrifugation, the supernatant was boiled with 2X SDS buffer, and the samples were loaded onto an SDS-page gel and transferred onto membranes. The membranes were then blocked with 5% skim milk and incubated with the primary antibody, followed by a secondary antibody. Protein detection was achieved using the ECL reagent solution. For IP, cell lysates were incubated with antibodies at 4 °C overnight on a rotator. Protein A/G PLUS-agarose beads were then added and incubated at 4 °C for 2 h on a rotator. The samples were washed with washing buffer and boiled with 2X SDS buffer at 100 °C. Ubiquitination and deubiquitination assays were performed by the ubiquitination assay kit according to the manufacturer’s manual (UBAK-100, D&P Biotech Inc., Seoul, Korea).

### Glutathione *S*-transferase (GST) pull-down assay

The GST pull-down assay was performed according to the previously described method [25]. In brief, the BL21 bacterial strain was transformed with GST vector (pGEX-4T-1) and GST-YOD1 and incubated overnight in Luria–Bertani broth (MB-L4488, KisanBio, Inc., Seoul, Republic of Korea) with 0.5 mM isopropyl β-d-1-thiogalactopyranoside at 18 °C. GST-YOD1 fastened Glutathione Sepharose was used to incubate the BL21 competent cells. The proteins bound to GST-YOD1 were washed with a washing buffer (0.1 M Tris–HCl at pH 7.4, 0.5 M NaCl, 20 mM imidazole at pH 7.4) and then separated by boiling with 2X SDS sample buffer. Western blotting was performed to detect the bound proteins.

### Immunocytochemistry (ICC)

The ICC procedure was performed according to the protocol as previously described [26]. Briefly, HEK293T cells were seeded on glass coverslips, placed on a 12-well plate, fixed with 4% formaldehyde, blocked, and incubated with primary antibodies (YOD1 and USP21) at 4 °C overnight. The cells were then washed with phosphate-buffered saline (PBS, P4417, Sigma-Aldrich, St. Louis, MO, USA), and incubated with Alexa-Fluor-488-conjugated goat anti-mouse (a11001, Invitrogen, Carlsbad, CA, USA) and with Alexa-Fluor-568-conjugated goat anti-rabbit (a11011, Invitrogen, Carlsbad, CA, USA). After washing with PBS, the cells were stained with DAPI (1 mg/ml stock, 1:1000, D9542, Sigma- Aldrich, St. Louis, MO, USA), and visualized using a confocal microscope (Zeiss LSM880, Carlz Zeiss Microscopy GmbH, Jena, Germany).

### Wound healing assay

HEK293T (1.2 × 10^6^), A549 (1.0 × 10^6^), and HeLa (1.0 × 10^6^) cells were seeded in 6-well plates 24 h after transfection of a mock control, Myc-*YOD1* alone, Flag-*USP21* alone, or both Myc-*YOD1* and Flag-*USP21*. A cell-free area was created by using a 10 µl pipette tip to scrape the cells, and the cell migration to the cell-free area was evaluated at 0, 12, 24, or 48 h using Image J software (National Institutes of Health, Bethesda, MD, USA).

### Cell counting kiy-8 (CCK-8) assay

For the CCK-8 assay, cells (3 × 10^3^) transfected with Myc-*YOD1*-, Flag-*USP21*-, or both Myc-*YOD1* and Flag-*USP21* were seeded into 96-well plates. After 0, 12, 24, or 48 h, the cells were incubated with CCK-8 (CK04, Dojindo, Kumamoto, Japan) in medium for 2 h, and optical density (OD) was measured at 450 nm using a microplate reader (Tecan Group Ltd. Seestrasse, Manndorf, Switzerland).

### Colony forming assay

For mock control, Myc-*YOD1*, Flag-*USP21*, or both Myc-*YOD1* and Flag-*USP21*-transfected cells (1 × 10^3^), 100-mm dishes were used for seeding. After 14 days, the cells were stained with crystal violet (27210‑0350, Junsei, Tokyo, Japan) to visualize colonies. Culture plates containing colonies were captured using a DUALED Blue/White Transilluminator (A-6020, Bioneer, Daejeon, Korea), and images were obtained. The number of colonies was counted using Image J (National Institutes of Health, Bethesda, MD, USA) after washing with PBS.

### Flow cytometry analysis

To synchronize cells at the G1/S boundary, we employed the double thymidine block method [27]. A549, HEK293T, and HeLa cells were transfected with a mock control, Myc-*YOD1* alone, Flag-*USP21* alone, or both Myc-*YOD1* and Flag-*USP21*. After transfection, the cells were fixed with 70% ethanol at 4 °C for 2 h. Subsequently, they were incubated with an anti-Ki67 antibody (sc-23900, Santa Cruz Biotechnology, Santa Cruz, CA, USA) at a dilution of 1:300 at room temperature for 30 min. This was followed by incubation with Alexa-Fluor-488-conjugated goat anti-mouse antibody (a11001, Invitrogen, Carlsbad, CA, USA) at a dilution of 1:300 at room temperature for 30 min. Finally, histogram analysis was performed using the CytoFLEX V0-B3-R1 Flow Cytometer (B53015, Beckman Coulter, Brea, CA, USA).

### Statistical analysis

The densitometric analysis was conducted using Image J software (National Institutes of Health, Bethesda, MD, USA). Statistical analysis including one-way ANOVA, two-way ANOVA, and paired *t*-test were performed using GraphPad Prism version 5 (GraphPad Software, La Jolla, CA, USA). Statistical significance was defined as *p < 0.05, **p < 0.01, ***p < 0.001. The presented results are representative data of at least three independent experiments and are expressed as mean ± standard error of the mean (SEM).

## Results

### YOD1 binds to USP21

**Fig. 1:**
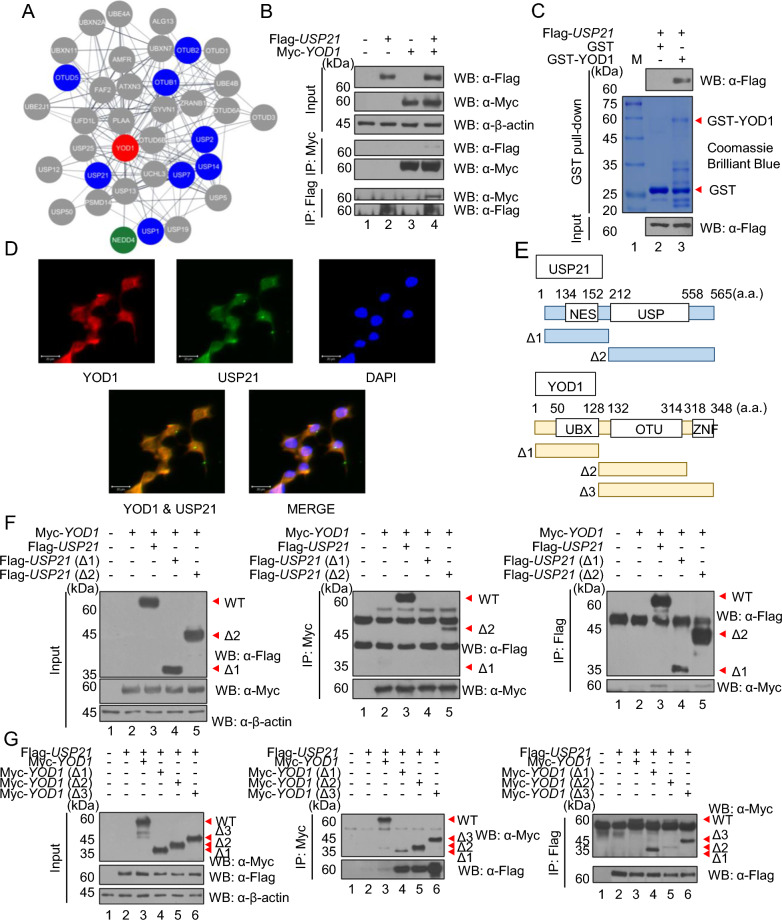
Identification of YOD1 substrates in the Hippo signaling pathway-related proteins using informatics tools. **A** A YOD1 interaction network was generated with STRING. The thickness of the line indicates the strength of association. The color of nodes indicates the confirmation of binding between YOD1 and proteins (gray, unpublished; blue, unpublished and related to the Hippo signaling pathway; and green, published and related to the Hippo signaling pathway). USP21 binding with YOD1 is shown in purple. **B** Myc-*YOD1* alone or in combination with Flag-*USP21* was transfected into HEK293T cells, IP with an anti-Flag or an anti-Myc antibody, followed by immunoblotting with antibodies against Myc and Flag. **C** Purified GST or GST-YOD1 was incubated with Flag-*USP21*-overexpressed HEK293T cell lysates. Purified GST and GST-YOD1 were visualized using Coomassie Brilliant Blue R/G staining solution. **D** ICC was performed to investigate the localization of respective YOD1 and USP21, and co-localization of YOD1 and USP21 (red, YOD1; green, USP21; and blue, DAPI). Scale bar, 20 μm. **E** Schematic representation of Flag-*USP21* and Myc-*YOD1* and their deletion mutants. **F** Myc-*YOD1* and Flag-*USP21* or its deletion mutants were co-transfected into HEK293T cells, followed by IP with an anti-Myc antibody and subsequent immunoblotting with Myc and Flag antibodies. **G** Flag-*USP21* and Myc-*YOD1* or its deletion mutants were co-transfected into HEK293T cells, followed by IP with an anti-Flag antibody, and subsequent immunoblotting with Myc and Flag antibodies

In a previous study, we identified that YOD1 regulates the Hippo signaling pathway by binding and deubiquitinating NEDD4 [22]. To investigate other additional substrates of YOD1, we utilized the bioinformatics tools, BioGRID, STRING, and Cytoscape. Due to the numerous putative substrates of YOD1, we narrowed our focus to those related to the Hippo signaling pathway and UPS. Among the putative substrates of YOD1, USP21 was found to be associated with both the Hippo signaling pathway and the UPS (Fig. 1A). Subsequently, we investigated the interaction between YOD1 and USP21, and our findings demonstrate that YOD1 indeed binds to USP21 (Fig. 1B, C). To assess their co-localization, an ICC assay was performed, revealing the co-localization of YOD1 and USP21 in both the nucleus and the cytoplasm (Fig. 1D). To identify the binding domain between YOD1 and USP21, we designed deletion mutant forms of both proteins (Fig. 1E), and performed a co-immunoprecipitation (co-IP) assay. Our results indicate that the C-terminal ubiquitin specific protease (USP) domain of USP21 is essential for its interaction with YOD1 (Fig. 1F). Furthermore, a co-IP assay between USP21 and deletion forms of YOD1 revealed that the ubiquitin regulatory X (UBX), ovarian tumor (OTU) domain of YOD1 are required for the interaction between USP21. Additionally, the zinc finger (Znf) domain was found to influence the binding affinity (Fig. 1G). Collectively, our findings suggest that YOD1 has the strong affinity to bind to USP21.

### Both YOD1 and USP21 are ubiquitinated

**Fig. 2:**
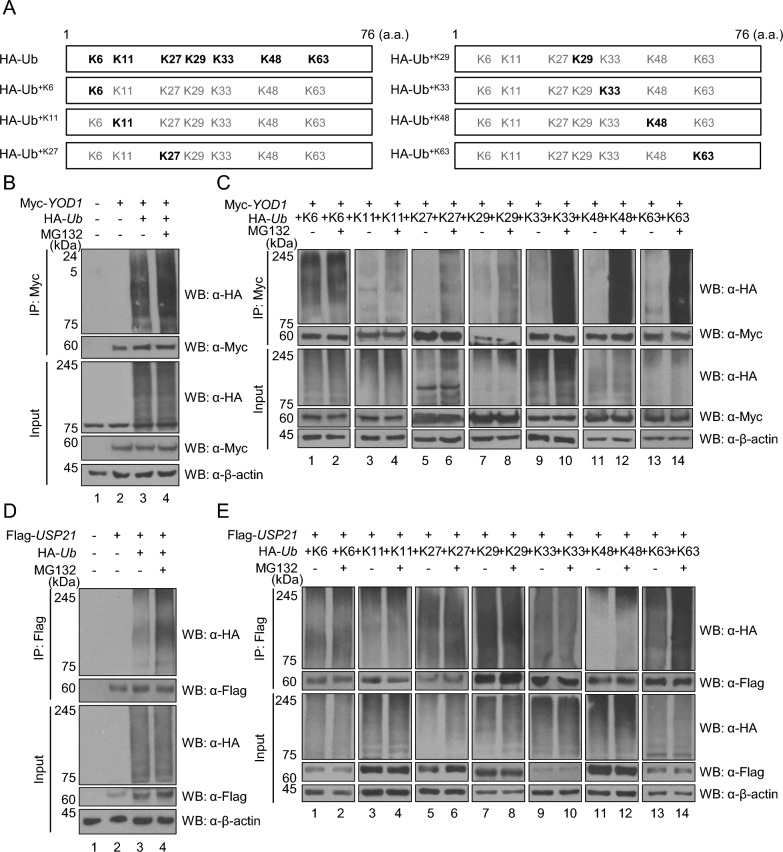
Ubiquitinated and regulation of YOD1 and USP21 by the UPS. **A** A diagram of lysine mutant constructs of ubiquitin. **B** A ubiquitination assay was performed on Myc-YOD1 by co-transfecting HEK293T cells with Myc-*YOD1* and HA-*Ub.* **C** HEK293T cells were transfected with *Myc-YOD1* and HA-*Ub* along with seven lysine mutant forms of ubiquitin (HA-*Ub*^+*K6*^, HA-*Ub*^+*K11*^, HA-*Ub*^+*K27*^, HA-*Ub*^+*K29*^, HA-*Ub*^+*K33*^, HA-Ub^+*K48*^, and HA-*Ub*^+*K63*^) to perform a more detailed analysis of Myc-YOD1. IP was conducted with an anti-Myc antibody, and the ubiquitination level was detected with an anti-HA antibody. **D** Flag-*USP21* was co-transfected with HA-*Ub* into HEK293T cells the ubiquitination assay. **E** A similar ubiquitination assay was conducted for Flag-USP21, with HEK293T cells being transfected with Myc*-YOD1* and HA-*Ub*, along with its seven lysine mutant forms of ubiquitin

In this study, we investigated the ubiquitination of Myc-YOD1 and Flag-USP21 using wild-type HA-*Ub* and various lysine mutant forms of it. To achieve this, we generated lysine mutant forms of HA-*Ub* by replacing all lysine residues with arginine, leaving only one specific lysine intact (including HA-*Ub*^+*K6*^, HA-*Ub*^+*K11*^, HA-*Ub*^+*K27*^, HA-*Ub*^+*K29*^, HA-*Ub*^+*K33*^, HA-*Ub*^+*K48*^, and HA-*Ub*^+*K63*^) (Fig. 2A). We also assessed the regulation of YOD1 by the UPS through the use of the proteasome inhibitor MG132 (Fig. 2B). Additionally, we examined which lysine-linked polyubiquitin chain is associated with the proteasomal degradation of Myc-YOD1. Surprisingly, each lysine mutant form of HA-Ub was capable of forming polyubiquitin chains on Myc-YOD1, and each type of lysine mutant form-linked polyubiquitin chain on Myc-YOD1 was related to proteasomal degradation (Fig. 2C). We then performed the ubiquitination assay on the UPS-regulated Flag-USP21 (Fig. 2D) and observed that an increase in the expressions of K11-, K27-, K48-, and K63-linked polyubiquitin chains on Flag-USP21 in MG132-treated cells (Fig. 2E). These results indicate that both YOD1 and USP21 undergo ubiquitination and are regulated by UPS.

### YOD1 and USP21 can deubiquitinate one another

**Fig. 3:**
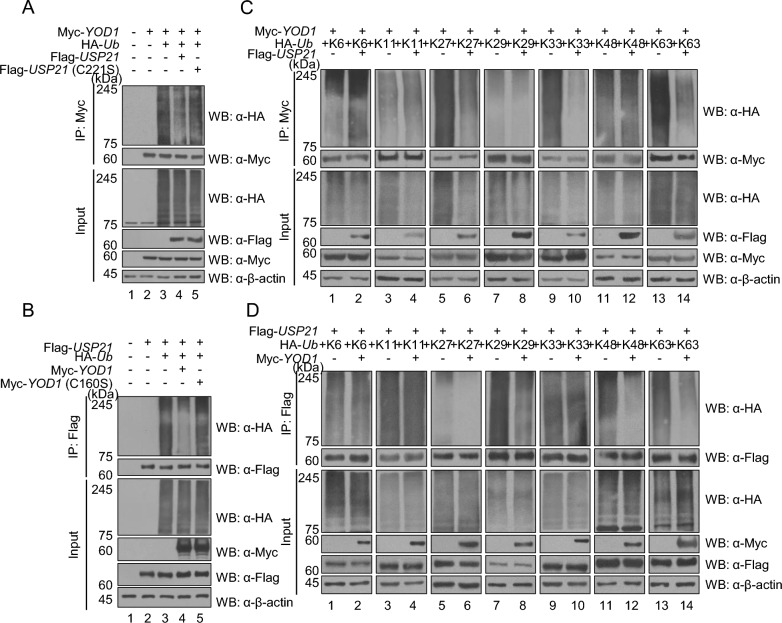
YOD1 and USP21 exhibit reciprocal deubiquitination activity on polyubiquitin chains. **A** The deubiquitination assay for Myc-YOD1: HEK293T cells were transfected with Myc-*YOD1,* Flag-*USP21*, and Flag-*USP21* (C221S) along with HA-tagged *Ub*. Myc-YOD1 was shown to deubiquitinate K27, K29, K48, and K63-linked chains on USP21. **B** The deubiquitination assay for Flag-USP21: HEK293T cells were transfected with Flag-*USP21*, Myc-*YOD1*, and Myc-*YOD1* (C160S) along with HA-tagged *Ub*. **C** The deubiquitination assay for Myc-YOD1: HEK293T cells were transfected with Myc-*YOD1,* Flag-*USP21* and HA tagged-ubiquitin constructs with specific ubiquitin mutants. **D** The deubiquitination assay for Flag-USP21: Flag-*USP21* was co-transfected with Myc-*YOD1* and HA tagged-specific mutant ubiquitin constructs into HEK293T cells

After identifying the binding between YOD1 and USP21, we aimed to investigate which protein acts as a DUB on the other. In the deubiquitination assay of Myc-YOD1, the formation of a polyubiquitin chain on Myc-YOD1 was reduced by Flag-USP21 compared to a catalytically inactive mutant of Flag-USP21 (C221S), indicating that USP21 functions as a DUB of YOD1 (Fig. 3A). Similarly, in the deubiquitination assay of Flag-USP21, the formation of a polyubiquitin chain on Flag-USP21 was reduced by Myc-YOD1 compared to a catalytically inactive mutant of Myc-YOD1 (C160S), indicating that YOD1 functions as a DUB of USP21 (Fig. 3B). To determine which type of polyubiquitin chain is regulated by USP21 on YOD1, we conducted a deubiquitination assay using specific ubiquitin constructs. We found that USP21 deubiquitinates K27-, K29-, K33-, and K63-linked polyubiquitin chains on YOD1 (Fig. 3C). Similarly, to identify which type of polyubiquitin chain on USP21 is regulated by YOD1, we performed a deubiquitination assay and observed that YOD1 deubiquitinates K27-, K29-, K48-, and K63-linked polyubiquitin chains on USP21 (Fig. 3D). These results indicate that YOD1 and USP21 function as DUBs of each other by catalyzing the removal of ubiquitin from specific polyubiquitin chains.

### YOD1 upregulates USP21 stability, but not vice versa

**Fig. 4:**
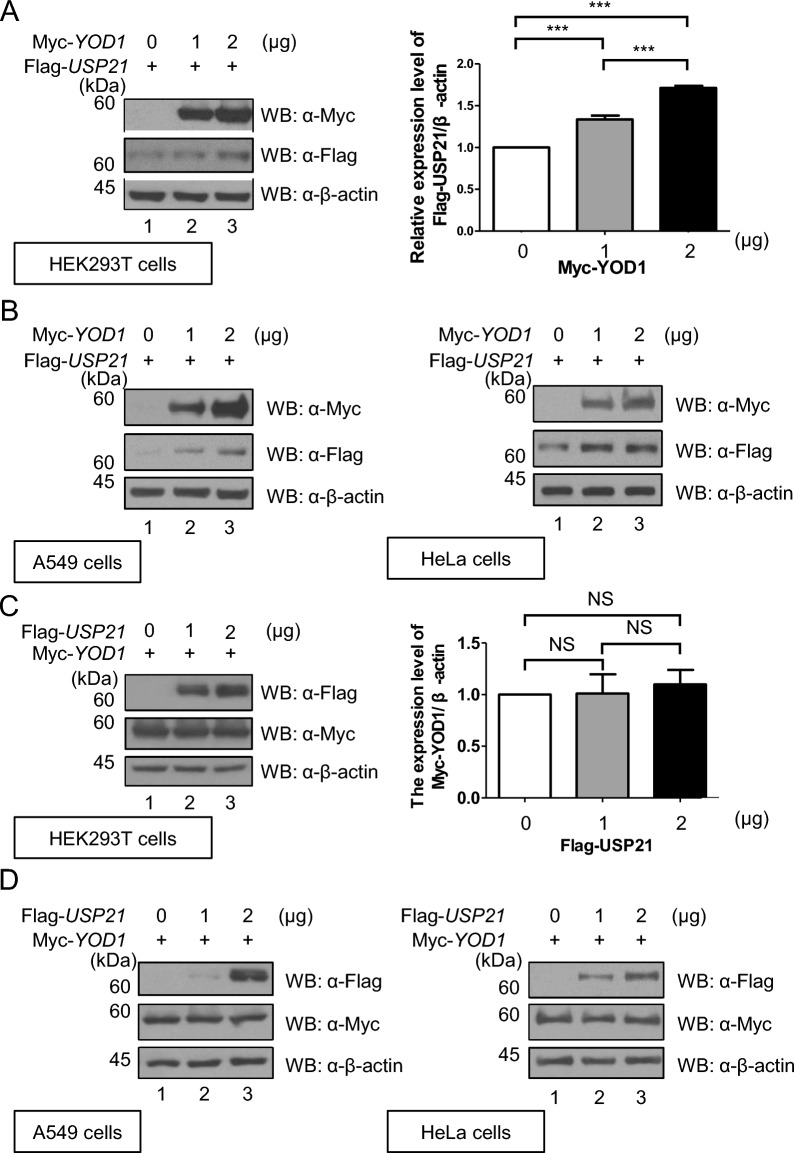
YOD1 upregulates USP21 protein stability, but not vice versa. **A** HEK293T cells were transfected with increasing amounts of Flag-*USP21* and Myc-*YOD1*. Western blotting was used to assess the expression level of Flag-USP21. The expression of Flag-USP21 was quantified in at three independent experiments using one-way ANOVA analysis and the data are presented as means ± SEM (with error bars) (***p < 0.001). **B** HeLa and A549 cells were transfected with increasing amounts of Flag-*USP21* and Myc-*YOD1*. Western blotting was used to assess the expression level of Flag-USP21. **C** HEK293T cells were transfected with increasing amounts of Myc-*YOD1* and Flag-*USP21*. Western blotting was used to assess the expression level of Myc-YOD1. The expression of Myc-YOD1 was quantified in at three independent experiments using one-way ANOVA and the data are presented as means ± SEM (with error bars) (*NS* not significant). **D** HeLa and A549 cells were transfected with increasing amounts of Myc-*YOD1* and Flag-*USP21*. Western blotting was used to assess the expression level of Myc-YOD1

In previous studies, polyubiquitin chains have been shown to trigger the proteasomal degradation of target substrates [17]. In the current study, we investigated the regulation of YOD1 and USP21 by the UPS and their ability to upregulate each other’s protein stability. To evaluate the effect of YOD1 on the stability of USP21, we transfected HEK293T, HeLa, and A549 cell lines with increasing doses of Myc-YOD1 and assessed the expression level of exogenous USP21. Our results demonstrated that YOD1 increased the stability of USP21 in all three cell lines (Fig. 4A, B). Subsequently, we transfected increasing doses of Flag-*USP21* into HEK293T, HeLa, and A549 cells to evaluate the stability change of Myc-YOD1. Interestingly, we found that the expression level of Myc-YOD1 remained unchanged in HEK293T cells exposed to Flag-*USP21* (Fig. 4C), and there was no significant difference in YOD1 stability observed in HeLa and A549 cells (Fig. 4D).

### Cellular synergistic effect of USP21 and YOD1 on cell proliferation

**Fig. 5:**
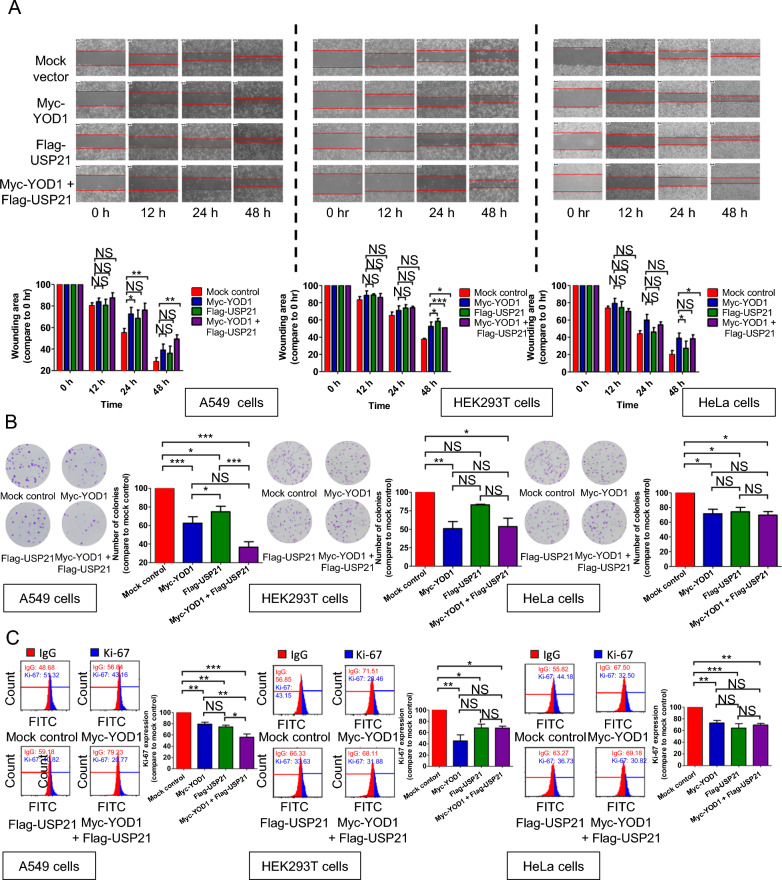
Cellular synergistic effect of USP21 and YOD1 on cell proliferation and Hippo signaling pathway. **A** Wound healing assays were conducted in Myc-*YOD1*-transfected cells alone or in combination with Flag-*USP21*-transfected cells. The wound area (%) was determined by calculating the remaining wound area compared to the control. The data are presented as means ± SEM (with error bars) from three independent experiments (*p < 0.05, **p < 0.01, ***p < 0.001, NS = not significant). Scale bars represent 100 μm. **B** Crystal violet staining was used to analyze the number of colonies in each of the transfected cell lines cultured in 100-mm dishes. The number of colonies was determined as the mean ± SEM (with error bars) from at least three independently repeated experiments (*p < 0.05, **p < 0.01, ***p < 0.001, *NS* not significant). **C** Flow cytometry analysis was conducted using Ki-67 staining to confirm cell proliferation in Myc-*YOD1*-transfected cells alone or in combination with Flag-*USP21*-transfected cells. The data are presented as means ± SEM (with error bars) from at least three independent experiments (*p < 0.05, **p < 0.01, ***p < 0.001, *NS* not significant)

We investigated the effects of USP21 and YOD1 on the cell proliferation, survival, and apoptosis since the Hippo signaling pathway is known to regulate these cellular functions [28]. To do this, we conducted wound healing, CCK-8, and colony forming assays in A549 cells. Our results showed that Flag-*USP21*-transfected cells exhibited slightly inhibited cell proliferation compared to a mock control, while Myc-YOD1-transfected cells showed inhibited cell proliferation. Additionally, co-transfection of Myc-*YOD1* and Flag-*USP21* into A549 cells resulted in even more inhibited cell proliferation (Fig. 5A, B). On the other hand, Myc-*YOD1*-transfected HEK293T and HeLa cells showed suppressed cell proliferation compared to a mock control, but no synergistic effect of USP21 and YOD1 was observed in these cell lines (Fig. 5A, B). In the CCK-8 assay results of A549, there was a synergistic effect of USP21 and YOD1, but no significant difference was observed in HEK293T or HeLa cells between the mock control and transfected groups (Additional file 1: Figure S1). We also performed flow cytometry analysis using Ki-67 staining, which is commonly used as a proliferation indicator, and the results were consistent with the findings mentioned above (Fig. 5C).

### Synergistic effect of USP21 and YOD1 on the Hippo signaling pathway

**Fig. 6:**
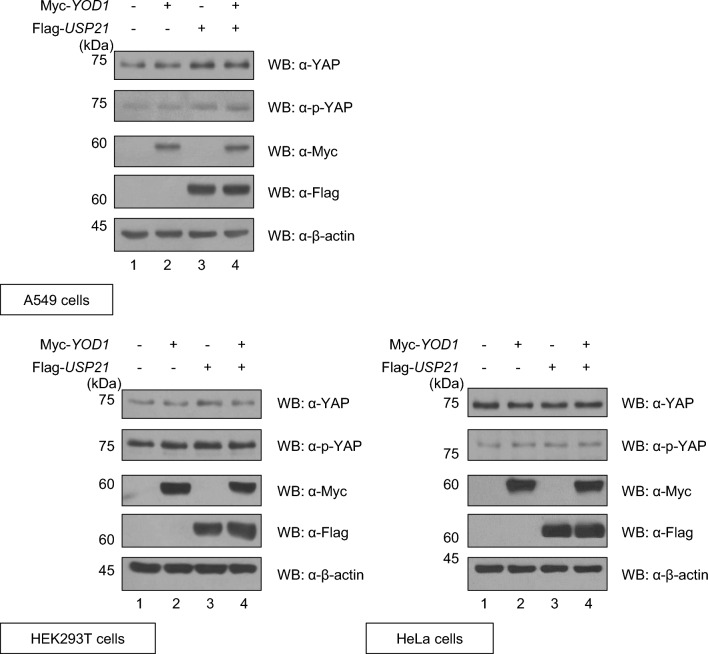
The cellular synergistic effect of USP21 and YOD1 on p-YAP. Western blotting was performed to determine the expressions of YAP and p-YAP in cells transfected with Myc-*YOD1* alone or in combination with Flag-*USP21*

Previous studies have reported that YOD1 and USP21 can independently increase the expression of p-YAP through the involvement of NEDD4 and MARK, respectively [22, 24]. In this study, we aimed to investigate whether the expression level of p-YAP is further increased through the synergic effect of YOD1 and USP21. We performed western blotting to assess this in A549 cells. We co-transfected Myc-*YOD1* and Flag-*USP21*, and found elevated levels of p-YAP compared to cells transfected with either Myc-*YOD1* or Flag-*USP21* alone (Fig. 6). However, we observed no synergistic effects of YOD1 and USP21 on the expression level of p-YAP in HEK293T or HeLa cells (Fig. 6). Our findings suggest that the combined action of YOD1 and USP21 inhibits A549 cell proliferation via the Hippo signaling pathway.

## Discussion

The Hippo signaling pathway plays a critical role in regulating cell proliferation to control organ growth [29]. Recent research conducted a systematic profiling of 9,125 tumor samples and identified widespread dysregulation of Hippo pathway components in various human cancer types, including colorectal cancer, endometrial cancer, and malignant glioma [2]. Over the years, many studies have investigated the regulation of YAP and TAZ by DUBs, either via Hippo-dependent or Hippo-independent mechanisms. Ubiquitin-specific peptidase 9X-linked (USP9X) interacts with LATS kinase and regulates YAP and TAZ activity [30]. Additionally, USP9X deubiquitinates AMOT, a YAP inhibitor, leading to the stabilization of AMOT and lower YAP/TAZ activity [31]. Meanwhile, ubiquitin-specific peptidase17 (USP17) regulates YAP/TAZ activity by deubiquitinating and stabilizing the E3 ligase ITCH. USP17 is also known to stabilize LATS kinases and AMOT proteins [32]. Several DUBs can regulate YAP/TAZ through Hippo-independent mechanisms. For instance, USP9X, ubiquitin-specific peptidase 47 (USP47), and OTU deubiquitinase ubiquitin aldehyde binding 1 (OTUB1) directly deubiquitinate YAP [33–35]. Furthermore, ubiquitin-specific peptidase 11 (USP11) deubiquitinates and upregulates vestigial like family member 4 (VGLL4), a transcriptional repressor that interacts with transcription factors TEADs. Knockdown of USP11 promotes cell proliferation, migration, and invasion in a YAP-dependent manner [36]. Regarding TAZ, ubiquitin-specific peptidase 1 (USP1), ubiquitin-specific peptidase 7 (USP7), ubiquitin-specific peptidase 26 (USP26), and ubiquitin-specific peptidase 36 (USP36) deubiquitinate TAZ [37–40]. Additionally, Josephin domain-containing protein 2 (JOSD2) and poly-SUMOylated OTU deubiquitinase ubiquitin aldehyde binding 2 (OTUB2) deubiquitinate and stabilize YAP/TAZ [41, 42].

In a previous study, we demonstrated that YOD1 regulates the Hippo signaling pathway by deubiquitinating NEDD4, an E3 ligase of LATS1 [23]. The interaction between YOD1 and NEDD4 was uncovered using the PPI network [22]. In this study, we aimed to identify potential substrates of YOD1 implicated in the Hippo signaling pathway. To accomplish this, we utilized the PPI database and bioinformatics tools (BioGRID, STRING, and Cytoscape) and discovered a cellular interaction between YOD1 and USP21 through co-IP, GST pull-down, and ICC assays (Fig. 1A–D). Notably, we observed that USP21 and YOD1 mutually deubiquitinate each other (Fig. 3A, B). We subsequently investigated how YOD1 and USP21 regulate each other’s protein stability. The ubiquitination assay of YOD1 revealed that wild-type and lysine mutant-linked polyubiquitin chains on YOD1 are associated with the proteasomal degradation of YOD1 (Fig. 2B, C), and USP21 deubiquitinates K27-, K29-, K33-, and K63-linked polyubiquitin chains on YOD1 (Fig. 3C). However, USP21 did not regulate YOD1 stability (Fig. 4C, D). Conversely, the overexpression of YOD1 increased USP21 protein stability in HEK293T, HeLa, and A549 cell lines (Fig. 4A, B).

There is conflicting research on the cellular functions of USP21 in the A549 cell line. Nguyen et al. reported that USP21 knocking down USP21 increases the activity of YAP/TAZ and cell proliferation in A549 cells [24]. However, another study reported that USP21 promotes non-small cell lung cancer (NSCLC) cell proliferation, migration, and invasion, including A549 cells, by deubiquitinating and stabilizing an oncoprotein, Yin Yang-1 (YY1) [43]. In the current study, we investigated the cellular functions of USP21 and the synergistic effect with YOD1 on cell proliferation. Our findings indicate that YOD1 inhibits cell proliferation in A549 cells. Interestingly, co-transfection of Myc-*YOD1* and Flag-*USP21* led to greater inhibition of cell proliferation as compared to transfection with Myc-*YOD1* or Flag-*USP21* alone (Fig. 5A–C). However, we also observed that there were cell lines in which there were no synergistic effects of YOD1 and USP21 on cell proliferation. In HEK293T or HeLa cells, co-transfection of Myc-*YOD1* and Flag-*USP21* did not inhibit cell proliferation compared to transfection with Myc-*YOD1* or Flag-*USP21* alone (Fig. 5A–C). In addition, we investigated cellular functions of YOD1 and USP21 on YAP and p-YAP. Our results show that A549 cells co-transfected with Myc-*YOD1* and Flag-*USP21* exhibited increased expression levels of p-YAP compared to transfection with either Myc-*YOD1* or Flag-*USP21* alone (Fig. 6). These findings suggest that the synergistic effect of YOD1 and USP21 can suppress specific cancer cell proliferation by regulating the Hippo signaling pathway (Figs. 5, 6).

Our study also sheds light on the interaction between YOD1 and USP21, demonstrating the possibility of deubiquitination of one DUB by another DUB. The interaction between DUBs is not well understood [44]. Our findings provide new insights into how a DUB may regulate cellular functions and signaling pathways by deubiquitinating another DUB. It is worth noting that further studies are required to elucidate the specific cellular functions of YOD1 and USP21 in relation to the modulation of lysine-linked polyubiquitin chains on each other. Moreover, considering that different cell lines may exhibit varying results, it is crucial to perform additional experiments in diverse cell lines to validate and generalize our findings.

## Conclusion

In summary, our study revealed several notable interactions between YOD1 and USP21. Specifically, we observed that YOD1 and USP21 have the ability to deubiquitinate each other, and that YOD1 can enhance the stability of USP21, while the reverse does not hold true. Moreover, our findings suggest that the combined activity of YOD1 and USP21 has a synergistic effect on the cell proliferation of A549 cells, and that this effect is mediated by their regulation of the Hippo signaling pathway. Overall, these results highlight the intriguing possibility that one DUB may modulate the activity of another DUB within the cellular system. Our study provides novel insights into the regulatory mechanisms of YOD1 and USP21 in the context of the Hippo signaling pathway, and sheds lights on their potential as therapeutic targets for cancer treatment.

### Supplementary Information

**Additional file 1: Figure S1.** Cellular synergistic effect of USP21 and YOD1 on A549 cell proliferation. The CCK-8 assay results are presented as bar graphs demonstrating the proliferative effects of YOD1 and USP21 on A549, HEK293T, and HeLa cells. Data are presented as the means ± SEM (error bars) from at least three independent experiments (**p < 0.01, ***p < 0.001, NS = not significant).

## Data Availability

The datasets used and/or analyzed during the current study are available from the corresponding author on reasonable request.

## Abbreviations

DUB: Deubiquitination enzyme
E1: Ubiquitin-activating enzyme
E2: Ubiquitin-conjugating enzyme
E3: Ubiquitin-protein ligase
IP: Immunoprecipitation
MARK: Microtubule-affinity regulating kinase
NEDD4: Neural precursor cell expressed developmentally down-regulated protein 4
PPI: Protein–protein interactions
TAZ: Transcriptional coactivator with PDZ-binding motif
USP21: Ubiquitin-specific peptidase 21
UPS: Ubiquitin–proteasome system
YAP: Yes-associated protein 1

## References

[1] Wu Z, Guan KL (2021). Hippo signaling in embryogenesis and development. Trends Biochem Sci.

[2] Sanchez-Vega F, Mina M, Armenia J, Chatila WK, Luna A, La KC, Dimitriadoy S, Liu DL, Kantheti HS, Saghafinia S (2018). Oncogenic signaling pathways in the cancer genome atlas. Cell.

[3] Zhou Y, Huang T, Zhang J, Cheng ASL, Yu J, Kang W, To KF (2018). Emerging roles of Hippo signaling in inflammation and YAP-driven tumor immunity. Cancer Lett.

[4] Kang W, Zhou Y, To KF (2017). The large tumor suppressor family: friend or foe?. J Thorac Dis.

[5] Piccolo S, Dupont S, Cordenonsi M (2014). The biology of YAP/TAZ: Hippo signaling and beyond. Physiol Rev.

[6] Streich FC, Lima CD (2014). Structural and functional insights to ubiquitin-like protein conjugation. Annu Rev Biophys.

[7] Komander D, Rape M (2012). The ubiquitin code. Annu Rev Biochem.

[8] Tracz M, Bialek W (2021). Beyond K48 and K63: non-canonical protein ubiquitination. Cell Mol Biol Lett.

[9] Elia AE, Boardman AP, Wang DC, Huttlin EL, Everley RA, Dephoure N, Zhou C, Koren I, Gygi SP, Elledge SJ (2015). Quantitative proteomic atlas of ubiquitination and acetylation in the DNA damage response. Mol Cell.

[10] Durcan TM, Tang MY, Perusse JR, Dashti EA, Aguileta MA, McLelland GL, Gros P, Shaler TA, Faubert D, Coulombe B (2014). USP8 regulates mitophagy by removing K6-linked ubiquitin conjugates from parkin. EMBO J.

[11] Matsumoto ML, Wickliffe KE, Dong KC, Yu C, Bosanac I, Bustos D, Phu L, Kirkpatrick DS, Hymowitz SG, Rape M (2010). K11-linked polyubiquitination in cell cycle control revealed by a K11 linkage-specific antibody. Mol Cell.

[12] Suresh B, Lee J, Kim H, Ramakrishna S (2016). Regulation of pluripotency and differentiation by deubiquitinating enzymes. Cell Death Differ.

[13] Zhou B, Zeng L (2017). Conventional and unconventional ubiquitination in plant immunity. Mol Plant Pathol.

[14] Yu Y, Zheng Q, Erramilli SK, Pan M, Park S, Xie Y, Li J, Fei J, Kossiakoff AA, Liu L (2021). K29-linked ubiquitin signaling regulates proteotoxic stress response and cell cycle. Nat Chem Biol.

[15] Yuan WC, Lee YR, Lin SY, Chang LY, Tan YP, Hung CC, Kuo JC, Liu CH, Lin MY, Xu M (2014). K33-Linked polyubiquitination of coronin 7 by Cul3-KLHL20 ubiquitin E3 ligase regulates protein trafficking. Mol Cell.

[16] Huang Q, Zhang X (2020). Emerging roles and research tools of atypical ubiquitination. Proteomics.

[17] Grice GL, Nathan JA (2016). The recognition of ubiquitinated proteins by the proteasome. Cell Mol Life Sci.

[18] Kwon YT, Ciechanover A (2017). The ubiquitin code in the ubiquitin-proteasome system and autophagy. Trends Biochem Sci.

[19] Dosa A, Csizmadia T (2022). The role of K63-linked polyubiquitin in several types of autophagy. Biol Futur.

[20] Poondla N, Chandrasekaran AP, Kim KS, Ramakrishna S (2019). Deubiquitinating enzymes as cancer biomarkers: new therapeutic opportunities?. BMB Rep.

[21] Kim Y, Kim W, Song Y, Kim JR, Cho K, Moon H, Ro SW, Seo E, Ryu YM, Myung SJ (2017). Deubiquitinase YOD1 potentiates YAP/TAZ activities through enhancing ITCH stability. Proc Natl Acad Sci U S A.

[22] Park JH, Kim SY, Cho HJ, Lee SY, Baek KH (2020). YOD1 Deubiquitinates NEDD4 involved in the Hippo signaling pathway. Cell Physiol Biochem.

[23] Salah Z, Cohen S, Itzhaki E, Aqeilan RI (2013). NEDD4 E3 ligase inhibits the activity of the Hippo pathway by targeting LATS1 for degradation. Cell Cycle.

[24] Nguyen HT, Kugler JM, Loya AC, Cohen SM (2017). USP21 regulates Hippo pathway activity by mediating MARK protein turnover. Oncotarget.

[25] Park HB, Hwang S, Baek KH (2022). USP7 regulates the ERK1/2 signaling pathway through deubiquitinating Raf-1 in lung adenocarcinoma. Cell Death Dis.

[26] Do HA, Baek KH (2022). Protein phosphatase 2A regulated by USP7 is polyubiquitinated and polyneddylated. Oncol Rep.

[27] Chen G, Deng X (2018). Cell Synchronization by double thymidine block. Bio Protoc.

[28] Yu FX, Zhao B, Guan KL (2015). Hippo pathway in organ size control, tissue homeostasis, and cancer. Cell.

[29] Ramaccini D, Pedriali G, Perrone M, Bouhamida E, Modesti L, Wieckowski MR, Giorgi C, Pinton P, Morciano G (2022). Some insights into the regulation of cardiac physiology and pathology by the Hippo pathway. Biomedicines.

[30] Toloczko A, Guo F, Yuen HF, Wen Q, Wood SA, Ong YS, Chan PY, Shaik AA, Gunaratne J, Dunne MJ (2017). Deubiquitinating enzyme USP9X suppresses tumor growth via LATS kinase and core components of the Hippo pathway. Cancer Res.

[31] Thanh Nguyen H, Andrejeva D, Gupta R, Choudhary C, Hong X, Eichhorn PJ, Loya AC, Cohen SM (2016). Deubiquitylating enzyme USP9x regulates Hippo pathway activity by controlling angiomotin protein turnover. Cell Discov.

[32] Nguyen HT, Kugler JM, Cohen SM (2017). DUB3 deubiquitylating enzymes regulate Hippo pathway activity by regulating the stability of ITCH, LATS and AMOT proteins. PLoS ONE.

[33] Pan B, Yang Y, Li J, Wang Y, Fang C, Yu FX, Xu Y (2020). USP47-mediated deubiquitination and stabilization of YAP contributes to the progression of colorectal cancer. Protein Cell.

[34] Li L, Liu T, Li Y, Wu C, Luo K, Yin Y, Chen Y, Nowsheen S, Wu J, Lou Z (2018). The deubiquitinase USP9X promotes tumor cell survival and confers chemoresistance through YAP1 stabilization. Oncogene.

[35] Yan C, Yang H, Su P, Li X, Li Z, Wang D, Zang Y, Wang T, Liu Z, Bao Z (2022). OTUB1 suppresses Hippo signaling via modulating YAP protein in gastric cancer. Oncogene.

[36] Zhang E, Shen B, Mu X, Qin Y, Zhang F, Liu Y, Xiao J, Zhang P, Wang C, Tan M (2016). Ubiquitin-specific protease 11 (USP11) functions as a tumor suppressor through deubiquitinating and stabilizing VGLL4 protein. Am J Cancer Res.

[37] Mussell A, Shen H, Chen Y, Mastri M, Eng KH, Bshara W, Frangou C, Zhang J (2020). USP1 regulates TAZ protein stability through ubiquitin modifications in breast cancer. Cancers (Basel).

[38] Li J, Dai Y, Ge H, Guo S, Zhang W, Wang Y, Liu L, Cheng J, Jiang H (2022). The deubiquitinase USP7 promotes HNSCC progression via deubiquitinating and stabilizing TAZ. Cell Death Dis.

[39] Tang J, Luo Y, Xiao L (2022). USP26 promotes anaplastic thyroid cancer progression by stabilizing TAZ. Cell Death Dis.

[40] Wang D, Li Z, Li X, Yan C, Yang H, Zhuang T, Wang X, Zang Y, Liu Z, Wang T (2022). DUB1 suppresses Hippo signaling by modulating TAZ protein expression in gastric cancer. J Exp Clin Cancer Res.

[41] Qian M, Yan F, Wang W, Du J, Yuan T, Wu R, Zhao C, Wang J, Lu J, Zhang B (2021). Deubiquitinase JOSD2 stabilizes YAP/TAZ to promote cholangiocarcinoma progression. Acta Pharm Sin B.

[42] Zhang Z, Du J, Wang S, Shao L, Jin K, Li F, Wei B, Ding W, Fu P, van Dam H (2019). OTUB2 promotes cancer metastasis via Hippo-independent activation of YAP and TAZ. Mol Cell.

[43] Xu P, Xiao H, Yang Q, Hu R, Jiang L, Bi R, Jiang X, Wang L, Mei J, Ding F (2020). The USP21/YY1/SNHG16 axis contributes to tumor proliferation, migration, and invasion of non-small-cell lung cancer. Exp Mol Med.

[44] Loch CM, Strickler JE (2012). A microarray of ubiquitylated proteins for profiling deubiquitylase activity reveals the critical roles of both chain and substrate. Biochim Biophys Acta.

