# Supplementary material for: Synergistic effect of YOD1 and USP21 on the Hippo signaling pathway
Source: Cancer Cell Int. 2023 Sep 24;23:209. doi: 10.1186/s12935-023-03078-3 (PMC10518088; doi:10.1186/s12935-023-03078-3)

Figure S1. Cellular synergistic effect of USP21 and YOD1 on A549 cell proliferation. The proliferative effects of YOD1 and USP21 on A549, HEK293T, and HeLa cells were evaluated using the CCK-8 assay. Bar graphs representing the results are presented, indicating the effect on cell proliferation. The data, shown as the means ± SEM (error bars), were obtained from at least three independent experiments (***p* < 0.01, ****p* < 0.001, NS=not significant).


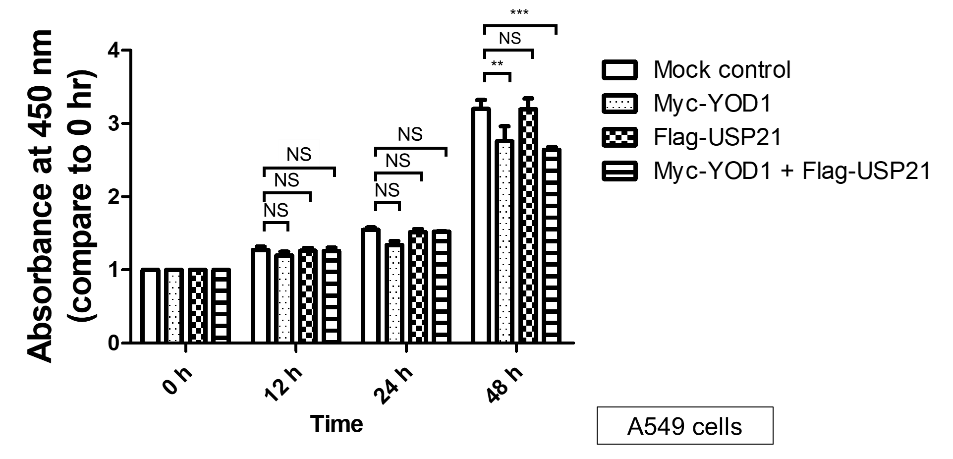


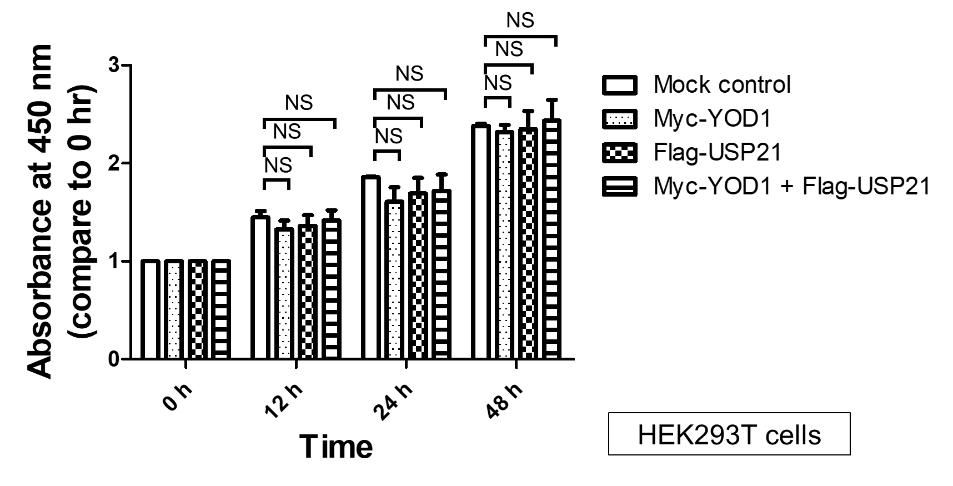


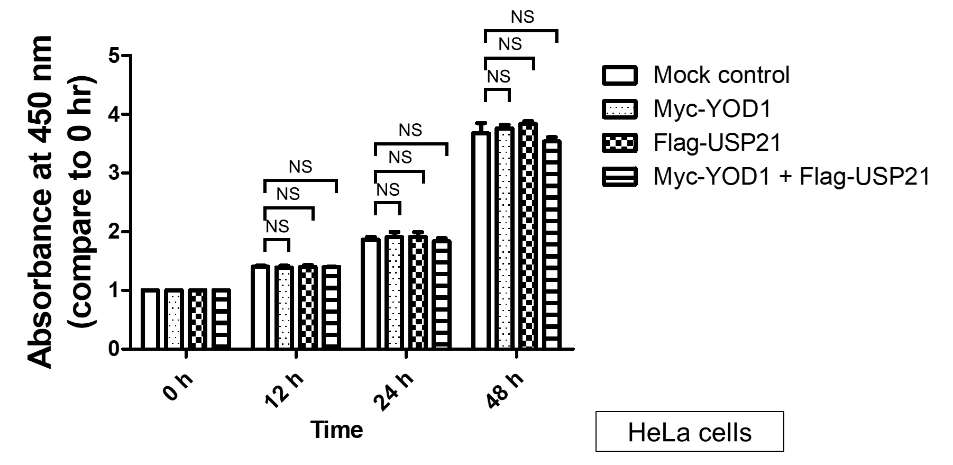

Supplement: Supplementary file 1 — Additional file 1: Figure S1. Cellular synergistic effect of USP21 and YOD1 on A549 cell proliferation. The CCK-8 assay results are presented as bar graphs demonstrating the proliferative effects of YOD1 and USP21 on A549, HEK293T, and HeLa cells. Data are presented as the means ± SEM (error bars) from at least three independent experiments (**p < 0.01, ***p < 0.001, NS = not significant). [file 12935_2023_3078_MOESM1_ESM.docx]
